# Feminization of the Isopod *Cylisticus convexus* after Transinfection of the *w*VulC *Wolbachia* Strain of *Armadillidium vulgare*

**DOI:** 10.1371/journal.pone.0128660

**Published:** 2015-06-05

**Authors:** Myriam Badawi, Pierre Grève, Richard Cordaux

**Affiliations:** Université de Poitiers, UMR CNRS 7267 Ecologie et Biologie des Interactions, Equipe Ecologie Evolution Symbiose, Bât. B8, 5 rue Albert Turpin, TSA 51106, 86073, Poitiers, Cedex 9, France; University of Innsbruck, AUSTRIA

## Abstract

Reproductive parasites such as *Wolbachia* are able to manipulate the reproduction of their hosts by inducing parthenogenesis, male-killing, cytoplasmic incompatibility or feminization of genetic males. Despite extensive studies, no underlying molecular mechanism has been described to date. The goal of this study was to establish a system with a single *Wolbachia* strain that feminizes two different isopod species to enable comparative analyses aimed at elucidating the genetic basis of feminization. It was previously suggested that *Wolbachia w*VulC, which naturally induces feminization in *Armadillidium vulgare*, induces the development of female secondary sexual characters in transinfected *Cylisticus convexus *adult males. However, this does not demonstrate that *w*VulC induces feminization in *C*. *convexus* since feminization is the conversion of genetic males into functional females that occurs during development. Nevertheless, it suggests that *C*. *convexus *may represent a feminization model suitable for further development. Knowledge about *C*. *convexus* sexual differentiation is also essential for comparative analyses, as feminization is thought to take place just before or during sexual differentiation. Consequently, we first described gonad morphological differentiation of *C*. *convexus *and compared it with that of *A*. *vulgare*. Then, *w*VulC was injected into male and female *C*. *convexus *adult individuals. The feminizing effect was demonstrated by the combined appearance of female secondary sexual characters in transinfected adult males, as well as the presence of intersexes and female biases in progenies in which *w*VulC was vertically transmitted from transinfected mothers. The establishment of a new model of feminization of a *Wolbachia* strain in a heterologous host constitutes a useful tool towards the understanding of the molecular mechanism of feminization.

## Introduction

Obligate intracellular bacteria exclusively replicate inside the cells of their host and they are predominantly transmitted through mother-offspring relationships [1]. As a consequence, some endosymbionts have adopted a strategy consisting of manipulating their host reproduction to maximise their transmission [2][3][4]. Indeed, reproductive parasite endosymbionts either favour the fitness of the infected females through cytoplasmic incompatibility (CI) [5][6], or induce sex-ratio biases towards females in host progenies [7] through male-killing (death of male progeny) [8][9], thelytokous parthenogenesis [10][11], or feminization of genetic males [12][13]. The alphaproteobacterium *Wolbachia* that infects many arthropod species and filarial nematodes is the only endosymbiont known to induce all four of these effects [14][15][16]. Considered the most widespread endosymbiont on the planet, *Wolbachia* infects at least 40% of insect species [16], some chelicerate species (mites, spiders and scorpions [17][18][19]), and crustaceans, in which at least 61% of terrestrial isopod species are infected [12][20].

In feminization induced by *Wolbachia*, host genetic sex determination is manipulated by the endosymbiont which converts genetic males into functional phenotypic females. *Wolbachia-*induced feminization is the most common phenotype observed in terrestrial isopod crustaceans [12][21][22][23], even if CI strains have been described in three species [24][25][26]. Despite extensive studies carried out since the discovery of *Wolbachia* as the feminizing agent of the isopod *Armadillidium vulgare* in the 1970s [22][27][28], no underlying molecular mechanism has been described to date [2].

In *A*. *vulgare*, embryos that inherited *Wolbachia* developed into functional females, in which bacteria prevented the development of the androgenic gland. This gland secretes the androgenic hormone responsible for the differentiation of primary and secondary male sexual characters [2][29]. *A*. *vulgare* gonad differentiation has been described in detail by Suzuki and Yamasaki [30] and it occurs within a period of ten to fifteen weeks after the release of juveniles from the female ventral pouch. Eight post-embryonic stages were defined, each corresponding to an intermolt stage. Gonads differentiate during stages 4 to 8 and androgenic glands progressively develop at the top of each testis from stage 6 to 8 [30]. *Wolbachia* is thought to act before or during sexual differentiation in order to inhibit male gonad differentiation, and convert genetic males into phenotypic females. When incomplete feminization occurs, this leads to intersexes ranging from sterile intersex males (iM) exhibiting female genital apertures and hypertrophied androgenic glands, to functional intersex females harboring typical male brushes on forelegs [31][32][33]. It has been hypothesized that this incomplete feminization is linked with low density of *Wolbachia* [34].

All these intersex phenotypes regularly observed in natural isopod populations can be experimentally produced by injection of *Wolbachia* in adult males. *Wolbachia*-induced iM may be obtained when the donor of the feminizing *Wolbachia* strain and the recipient belong to the same species [12][35][36]. In the case of interspecific transfers of *Wolbachia* in terrestrial isopod hosts, the efficiency of the feminizing strains decreases with phylogenetic distance of the recipient, leading to *Wolbachia* elimination, an absence of effect, death of the recipient or conservation of the effect [12][34][35][36]. It has been shown that the feminizing strain of *A*. *vulgare*, *w*VulC, is able to induce the development of female secondary sexual characters in *Cylisticus convexus* adult males [35], whereas the CI-inducing strain of *C*. *convexus*, *w*Con, is able to induce CI when transinfected to *A*. *vulgare* [25]. These observations showed that the *Wolbachia*-induced phenotypes are due to the injected strains and do not depend on the genetic background of the hosts. *C*. *convexus* may therefore be a suitable model to uncover the mechanisms of feminization. Hence, it would be relevant to compare feminization induced by the same strain of *Wolbachia*, *w*VulC, in two distinct host species. However, induction of female secondary sexual characters by *w*VulC in *C*. *convexus* adult males does not necessarily mean that there is feminization of *C*. *convexus*, as feminization is the conversion of the male genetic sex into a fully functional female. Moreover, since feminization is supposed to take place before or during sexual differentiation, it is essential to distinguish between the feminizing action of the bacteria linked to sexual differentiation and an alternative action of the bacteria unrelated to sexual differentiation (*i*.*e*., without any link to feminization) but fortuitously happening during sexual differentiation. Such a confounding effect may be uncovered by analyzing a feminizing *Wolbachia* strain in two distinct genetic backgrounds having different timings of sexual differentiation.

In this study, we first described the developmental stages of *C*. *convexus* by comparison with those of *A*. *vulgare* and identified a one-stage shift of sex differentiation timing between the two species. *w*VulC from *A*. *vulgare* was also injected into uninfected *C*. *convexus* adult females and males to test whether: (i) *w*VulC can be vertically transmitted from mother to offspring, and (ii) *w*VulC induces feminization in its new host (feminization being assessed by the appearance of female secondary sexual characters in adult males, as well as the presence of intersexes and female bias in transinfected female progenies). Altogether, our results provide formal demonstration of a feminizing effect of *w*VulC in *C*. *convexus*.

## Materials & Methods

### Transinfection of the *w*VulC Feminizing *Wolbachia* Strain in *C*. *convexus*

Isopods were reared at 20°C with food *ad libitum* (dead lime tree leaves and carrots) under natural photoperiod, except those in cross-breeding and juveniles which were reared under a 18L:6D photoperiod. Uninfected 10 month-old *C*. *convexus* males and females (from our laboratory line AW, derived from individuals caught in Villedaigne, France, in 1997) were infected by *w*VulC. The solution containing *Wolbachia* was prepared using ovaries from five *A*. *vulgare* females naturally harbouring the *w*VulC strain (from our laboratory line ZN, derived from individuals caught in Celles sur Belle, France, in 1991). The presence of *w*VulC was specifically checked by PCR and sequencing of the *wsp* sequence as described in Cordaux *et al*. [21]. Ovaries were crushed in 500 μL of Ringer solution (NaCl 394 mM; KCl 2 mM; CaCl_2_, 2H_2_O 2 mM; NaHCO_3_ 2 mM), and the resulting suspension was filtered through a 5 μm pore membrane (Sartorius Stedim Biotech). One microliter of the solution diluted twice with Ringer solution was directly injected in the general body cavity of *C*. *convexus* animals through a small hole pierced in each individual’s cuticle using a thin glass needle. A total of 16 males and 37 females were transinfected with *w*VulC. As controls, 6 males and 20 females were injected with Ringer solution only. After 6 months, the 49 surviving females (33 treated animals and 16 controls) were cross-bred with uninfected males. At birth, juveniles were separated from the parents and at three months old, males and females were separated. Fourteen months after injection, the 11 surviving males were dissected in Ringer solution, and sexual characters were directly examined under binocular magnifier (50X); including gonad morphology, male copulating pleopods, size of uropods (larger in males than in females), male brushes on pereiopods, and female genital apertures. In order to improve the contrast of the image (especially for gonads which are white), dark-field was used.

Finally, 14 months after injection, total DNA of gonads of all surviving animals (treated and control males and females) and gonads, nervous cords, and head tissues of all progenies were extracted using the Qiagen DNeasy Blood and Tissue kit according to the manufacturer's instructions. Before DNA extraction, gonads of all progenies were examined under binocular magnifier (50X) as described above. Success of DNA extractions was checked by PCR amplification of the mitochondrial marker COI according to Folmer *et al*. [37]. The presence of *Wolbachia* was tested by PCR amplification of at least two of three bacterial genes (*wsp*, *ftsZ* or *recR*) using *Wolbachia*-specific primers, according to Badawi *et al*. [38], Braig *et al*. [39], and Werren *et al*. [40].

### Developmental Study and Microscopy Observations

*C*. *convexus* females and males from our uninfected AW laboratory line were cross-bred. At birth, juveniles were separated from their parents. Sexual development of *C*. *convexus* was investigated in offspring of uninfected females and *w*VulC-transinfected females (obtained as described above). Molts were checked every 2 or 3 days from birth until stage 7. Ventral calcium white plates and evacuation of gut content occur a few days before each molt, which allows accurate developmental stage identification. Gonads of juveniles (3–14 individuals for each stage) were harvested right after molts for microscopic observations. Each gonad was fixed for 2h in a fixative solution (9% glutaraldehyde, 0.3 M sodium cacodylate, 3% NaCl; 1/1/1). Gonads were then washed for 1h in PBS solution and for 10 minutes in bi-distilled sterile water. Tissues were mounted between slide and slip cover in a citifluor drop before bright-field light microscope observation (200X for stage 1–3 gonads and 100X for stage 4–7 gonads). Photographs were taken as mosaics with ZEN software (ZEISS) when the object was larger than the microscopic field. Images were reconstructed with ZEN.

### Statistical Tests

The proportion of females and intersexes (F+I) was calculated for each brood. To estimate a bias towards the proportion of F+I considering animals of all progenies, we compared with a χ^2^ test the observed frequencies of males and F+I of infected and uninfected individuals with the mean frequency observed in the controls. We also compared for each type of clutch (clutch with infected individuals and clutch with no infected individuals) the total frequencies of males and F+I with the mean frequency observed in the controls.

## Results

### Sexual differentiation of *C*. *convexus*

We investigated gonad differentiation from birth to gonad maturity in 43 *C*. *convexus* individuals produced by uninfected females. The development was followed during the first 7 molts (17 weeks post-birth) and used to define post-embryonic developmental stages. Among the 29 individuals for whom the sex could be assessed (15 females and 14 males), there was a balanced sex-ratio (51.7% females; χ^2^ = 0.0345 df = 1; p = 0.85).

A few hours after birth, juveniles experienced a first ecdysis molt (defining start of stage 1). Duration of the following intermolts ranged from 2 to 3 weeks. Until the end of stage 2, juveniles were still mancas since they harbored only 6 pairs of functional pereiopods. At stage 2, the 7th pereiopod pair developed but remained non-functional and folded down the ventral face. Finally, stage 3 marked the first stage of larvae, as the 7th pereiopod pair was functional. Female genital apertures and male copulating pleopods were visible at stage 5. Other secondary sexual characters such as foreleg brushes or longer uropods in males developed after stage 7.

**Fig 1:**
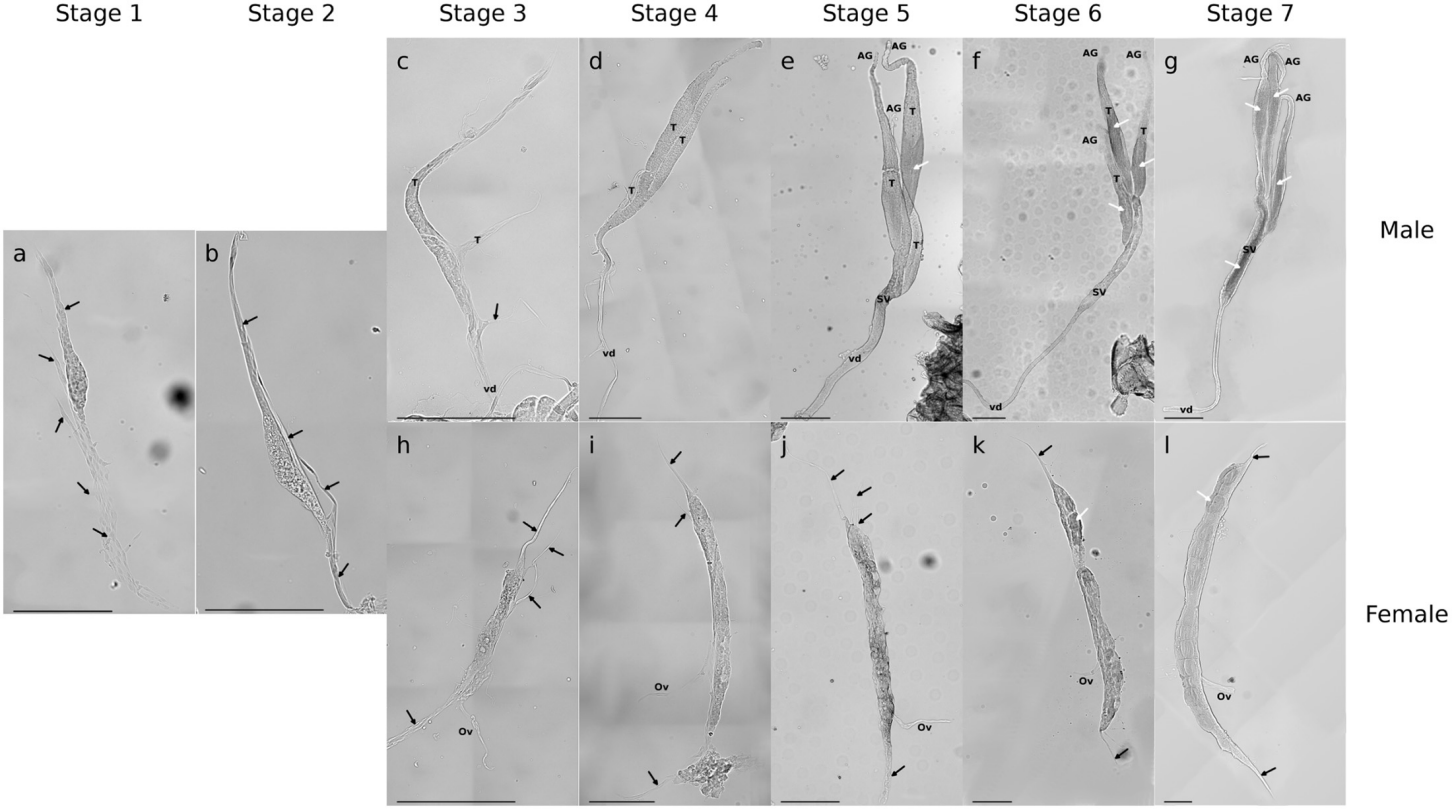
Micrographs of gonad morphology during post-embryonic development of *C*. *convexus* observed by light microscopy (a-c, h: 200X; d-g, i-k: 100X). Undifferentiated gonad during stage 1 (a) and stage 2 (b); differentiating male gonad during stage 3 (c), stage 4 (d), stage 5 (e); maturating male gonad during stage 6 (f), stage 7 (g); differentiating female gonad during stage 3 (h), stage 4 (i), stage 5 (j); maturating female gonad during stage 6 (k), stage 7 (l). T = testis, SV = seminal vesicle, AG = androgenic gland, vd = *vas deferens*, Ov = oviduct. Suspensory filaments are indicated with black arrows. Spermatogenesis and oogenesis are indicated with white arrows. The scale bar represents 200 μm.

Individuals from each stage were also dissected to follow gonad development. During stages 1 and 2, no distinct morphological difference between male and female gonads was observed. Juvenile gonads constituted undifferentiated tubes slightly swollen in the middle and harboring suspensory filaments from which testis and *vas deferens* in males, or oviduct in females will differentiate (Fig 1a and 1b). Stage 3 began ~30 days after birth and marked the start of gonad differentiation. In the male gonad, the first suspensory filament at the top of the gonad was elongating into testis, whereas the second one was just starting to differentiate. At the opposite end, the gonad was elongating into *vas deferens*. The central swollen region also tended to elongate (Fig 1c). At stage 4, two weeks later, the first two testis were fully formed whereas the third one was still differentiating (Fig 1d). At stage 5, two weeks later, testis 1 and 2 now harbored an androgenic gland. At the bottom of the gonad, the seminal vesicle was still differentiating. The onset of spermatogenesis was also observable in the first testis (Fig 1e). After three more weeks, at stage 6, spermatogenesis started in the three testis, which all presented an androgenic gland at their top. The seminal vesicle was now fully differentiated, as the lumen (still empty) was visible along its full length (Fig 1f). Finally, at stage 7, sperm filled the seminal vesicle and *vas deferens* (Fig 1g).

In females, at stage 3, the oviduct was differentiating and the gonad was elongating (Fig 1h and 1i). From stage 4, other suspensory filaments regressed (Fig 1i). Then, at stage 5, the very beginning of oogenesis was observed in the upper part of the gonad (Fig 1j). At stage 6, the second and third suspensory filaments almost disappeared and the posterior suspensory filament was regressing (Fig 1k). Finally, at stage 7, gonads continued to mature and newly developed oocytes were arranged in one line along the longitudinal axis of the ovary (Fig 1l).

### *w*VulC-Induced Feminization of *C*. *convexus* Adult Males

Bouchon *et al*. [35] showed that injection of *w*VulC into *C*. *convexus* adult males induced the formation of female genital apertures and hypertrophied androgenic glands after 14 months (which correspond to iM observed in *A*. *vulgare* males transinfected with *w*VulC). While this observation led the authors to suspect a feminizing effect of *w*VulC in *C*. *convexus*, it does not constitute a formal demonstration of a feminizing effect which requires: (i) transmission of the bacteria to the next host generation and, (ii) full conversion of genetic male individuals into functional females during development.

**Fig 2:**
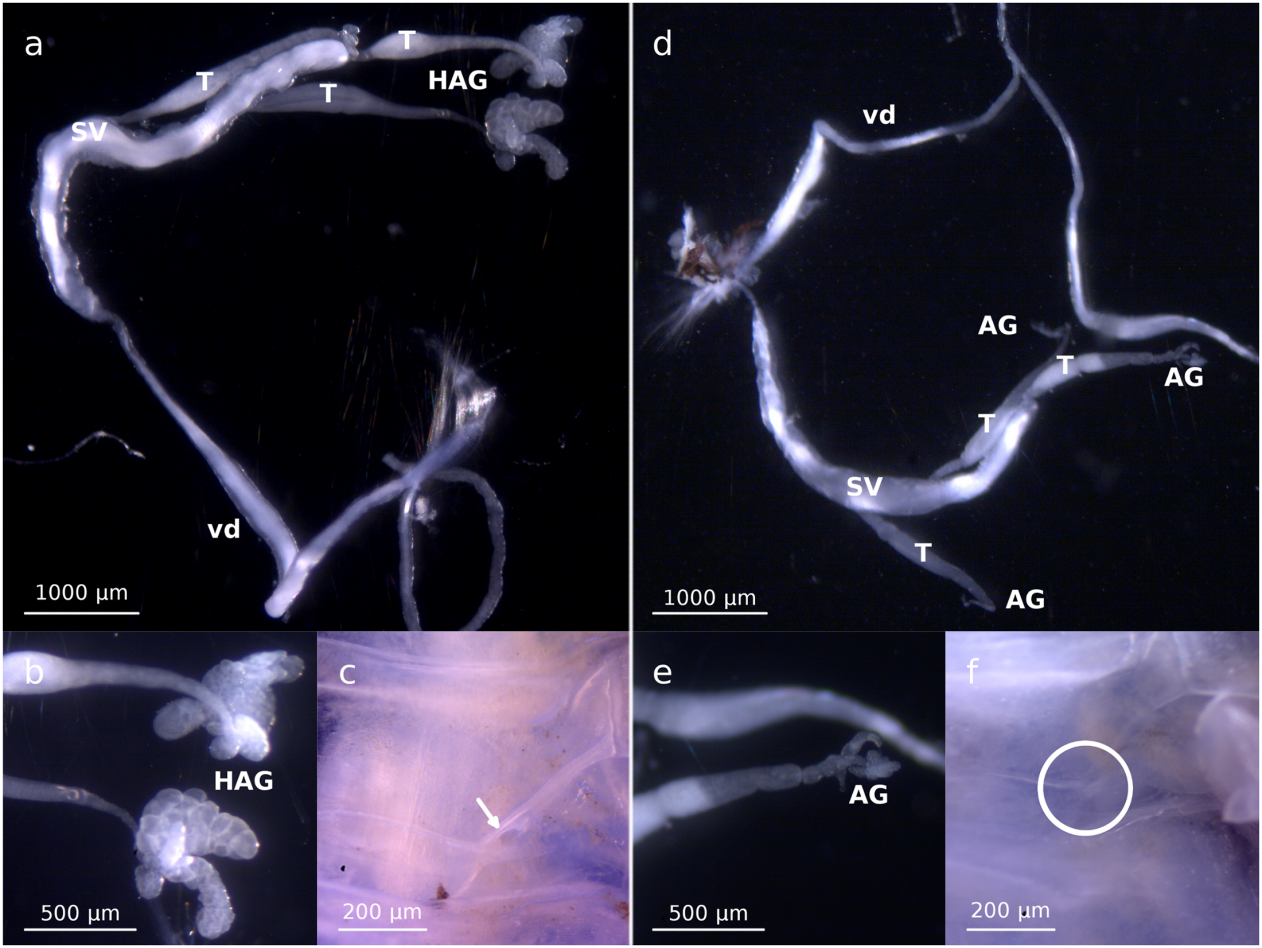
Gonad morphology of transinfected adult *C*. *convexus* males observed with binocular magnifiers (50X). (a) With magnification of the hypertrophied androgenic glands (b) and a genital aperture on the ventral face (indicated with a white arrow, at c). As a control, gonad morphology of Ringer-injected *C*. *convexus* males (d) with magnification of a normal androgenic gland (e) and the absence of genital apertures (highlighted by a white circle, at f). T = testis, SV = seminal vesicle, HAG = hypertrophied androgenic gland, vd = *vas deferens*, AG = androgenic gland.

First, we injected *w*VulC into *C*. *convexus* adult males, as did Bouchon *et al*. [35]. The difference was that while the recipient individuals used in Bouchon *et al*. [35] originated from a *C*. *convexus* line infected with the *w*Con *Wolbachia* strain, here we used a *Wolbachia*-free *C*. *convexus* line, to exclude any putative interaction between the transinfected *w*VulC strain and the native *w*Con strain that may have contributed to the observations made by Bouchon *et al*. [35]. Consistent with the observations by Bouchon *et al*. [35], 10 of 11 *w*VulC-transinfected *C*. *convexus* males exhibited hypertrophied androgenic glands (Fig 2a and 2b), and 6 of them harbored one (2 individuals) or two (4 individuals) female genital apertures (Fig 2c; Table 1). Other male sexual characters, such as the size of uropods, copulating pleopods, and pereiopod brushes were not altered. *Wolbachia* was detected by PCR in 9 of the 10 transinfected males exhibiting an intersexual phenotype (Table 1). All males harboring female genital apertures were infected by *Wolbachia*. *Wolbachia* was not detected in any of the controls, which exhibited all expected male sexual characters (Fig 2d,2e and 2f; Table 1).

**Table 1:** Characteristics of transinfected *C*. *convexus* adult males.

Treatment	AG^a^	fga^b^	*Wolbachia* ^c^	2nd Pr^d^	Ur1 (μm)^e^	Ur2 (μm)^e^	Pl (μm)^e^	Telson (μm)^e^
*w*VulC	H	2	+	brush	1092	2206	1332	3799
	H	2	+	brush	1852	1893	1231	3724
	H	2	+	brush	2344	2293	1461	3812
	H	2	+	brush	856	779	1428	3668
	H	1	+	brush	2441	2392	1302	3655
	H	1	+	brush	2540	NA	1279	3738
	H	0	+	brush	1611	2135	1180	3741
	H	0	+	brush	2453	2067	1043	3491
	H	0	+	brush	2079	1933	1131	3928
	H	0	-	brush	2727	2509	1322	3781
	N	0	-	brush	2229	2125	973	3257
Ringer	N	0	-	brush	2465	2299	963	3316
	N	0	-	brush	2373	1367	942	3272
	N	0	-	brush	2071	2176	972	3274
	N	0	-	brush	NA	NA	NA	NA

^a^ AG = Androgenic gland; N = Normal androgenic gland; H = Hypertrophied androgenic gland

^b^ Number of female genital apertures (fga)

^c^ Presence of *Wolbachia* detected by PCR using two molecular markers: *wsp* and recR.

^d^ Presence of brush on the second pair of pereiopods (Pr)

^e^ Size of uropods (Ur1 and Ur2), pleopod 2 (Pl) and telson

### Vertical Transmission of *w*VulC, Sex-Ratio Bias and Intersexes in *C*. *convexus*

To investigate whether *w*VulC can be vertically transmitted in *C*. *convexus*, *w*VulC was injected in adult females originating from the same *Wolbachia*-free *C*. *convexus* line used in the previous experiments. Vertical transmission of *w*VulC to offspring of 10 transinfected mothers, whose infection was confirmed by PCR, was investigated by PCR testing of all 159 offspring (64 males, 93 females and 2 intersexes based on external sexual characters) once they were adult (*i*.*e*., more than 6 months old). *w*VulC was detected in 25 individuals; 21 of which presented a female phenotype, 3 a male phenotype and 1 an intersex phenotype (Table 2).

**Table 2:** Vertical transmission of *w*VulC in *C*. *convexus* and proportion of females and intersexes (F+I).

Treatment	*Wolbachia* ^a^	M	F	I	Total	Proportion F+I
mother *w*VulC +;	3F	0	3	0	3	1.00
offspring with at least 1 *w*VulC +	1F	0	8	1	9	1.00
	7F; 1I	1	12	4	17	0.94
	5F	5	15	0	20	0.75
	3M; 4F	5	9	1	15	0.67
	1F	6	11	0	17	0.65
**Total**	**3M; 21F; 1I**	**17**	**58**	**6**	**81**	**0.79**
mother *w*VulC +;		6	11	7	24	0.75
offspring *w*VulC-		11	18	1	30	0.63
		6	5	0	11	0.45
		10	1	2	13	0.23
**Total**		**33**	**35**	**10**	**78**	**0.58**
mother *w*VulC-;		11	22		33	0.67
offspring *w*VulC-		15	26		41	0.63
		17	20		37	0.54
		11	13		24	0.54
		11	12		23	0.52
		18	18		36	0.50
		11	8		19	0.42
		9	3		12	0.25
**Total**		**103**	**122**		**225**	**0.54**

^a^ Presence of *Wolbachia* detected by PCR using two molecular markers: *wsp* and *ftsZ*.

F = Female, M = Male, I = Intersex.

**Fig 3:**
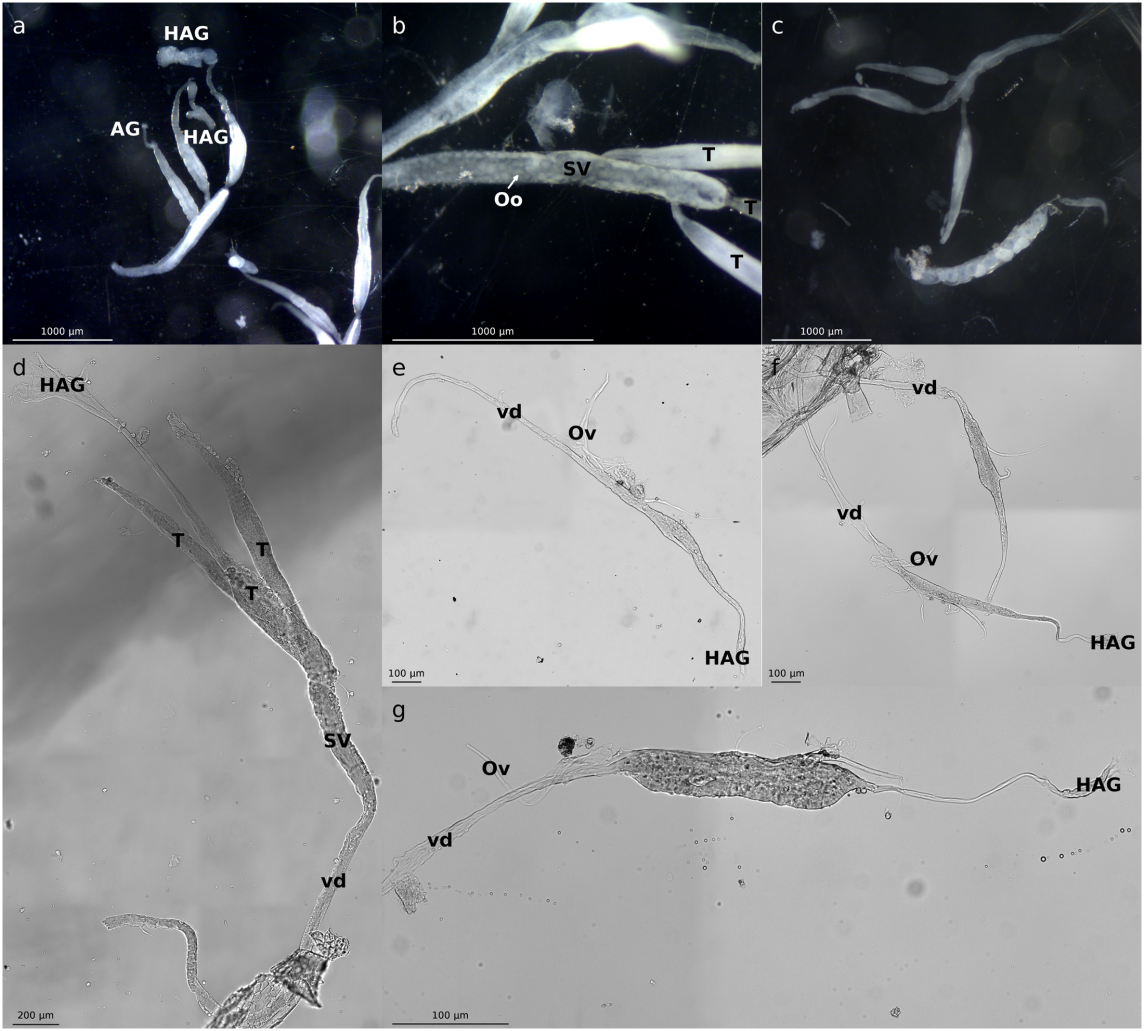
Gonad intersexual morphology of progenies produced by transinfected *C*. *convexus* females. Observed with binocular magnifiers in adult progenies (50X) (a-c) and by light microscopy during sexual differentiation (100X: d; 200X: e-g) at stage 3 (e), stage 5 (d,f) and stage 6 (g). HAG = hypertrophied androgenic gland, AG = androgenic gland, Oo = oocyte, SV = seminal vesicle, T = testis, vd = *vas deverens*, Ov = oviduct.

Strikingly, after dissection of the 159 individuals, we found that 14 individuals initially scored as males based on external sexual characters actually exhibited an intersex phenotype at the gonad level, harboring a whole gradient of male and female gonad morphology such as: male-like gonads with hypertrophied androgenic glands (Fig 3a), male-like gonads with oocytes in the seminal vesicle (Fig 3b), and co-occurence of both male-like and female like gonads within the same individual (with both male copulating pleopods and female genital apertures in two individuals; Fig 3c).

With respect to the 159 offspring from the 10 *w*VulC transinfected *C*. *convexus* mothers, the overall proportion of F+I was 68.6%, which was significantly higher (Χ^2^ = 13.16; df = 1; p<10^-3^) than in the 225 controls (54.2%) (Table 2). When considering the 25 individuals in which *w*VulC was detected by PCR, the proportion of F+I was 88.0%, which was significantly higher than in the 134 individuals in which *w*VulC was not detected (64.9%; Χ^2^ = 5.85; df = 1; p = 0.016) or the 225 controls (54.2%; Χ^2^ = 11.49; df = 1; p<10^-3^). Moreover, the proportion of F+I in the 6 broods in which *w*VulC was detected in at least one individual (79.0%) was significantly higher than in the 4 broods in which *w*VulC was not detected (57.7%; Χ^2^ = 15.08; df = 1; p<10^-3^) or in the 8 control broods (54.2%; Χ^2^ = 20.05; df = 1; p<10^-3^) (Table 2). The offspring from the controls presented a balanced sex-ratio (54.2% females, no intersex; Χ^2^ = 1.60; df = 1; p = 0.21).

To further investigate the feminizing action of *w*VulC in *C*. *convexus* during early development, gonads of 36 juveniles from a set of 5 *w*VulC-transinfected mothers were dissected during developmental stages 3 to 7. The number of post-embryonic molts was similar for juveniles produced by transinfected mothers compared to juveniles produced by uninfected mothers. Among the 36 dissected juveniles, 6 males and 16 females exhibited normal gonads, while the remaining 14 individuals were intersexes harboring altered gonads, ranging from unusual morphology (hypertrophy of androgenic glands; Fig 3d) to intersexual morphology (co-development of spermiduct and oviduct; Fig 3e,3f and 3g). Such alterations in gonad morphology were never observed in juveniles produced by uninfected mothers. As for adults produced by transinfected *C*. *convexus* females, we observed a significant bias towards F+I in juveniles (Χ^2^ = 13.3; df = 1; p<10^-3^) produced by transinfected mothers (83.3%) compared with the juveniles produced by uninfected mothers for which we also investigated gonad morphology during development (51.7%).

## Discussion

### Comparison of Sexual Differentiation Timing between *C*. *convexus* and *A*. *vulgare*

*C*. *convexus* gonad morphology is the same as that observed in several Oniscidea, including *A*. *vulgare* [30][41]. Indeed, male gonads present three testes, each surmounted by an androgenic gland. Each testis develops from suspensory filaments inserted onto the seminal vesicle, which extends by the *vas deferens* [41]. The duration of *C*. *convexus* embryonic development (~17 weeks), from marsupium release to stage 7, is apparently longer than that of *A*. *vulgare* (~10 weeks, according Suzuki and Yamasaki [30]). However, absolute duration is not directly comparable between the two studies. This is because post-embryonic development depends on rearing conditions such as temperature, which was different between the *A*. *vulgare* experiments (25°C) [30] and our study (21°C). Using our rearing conditions, *A*. *vulgare* post-embryonic development lasts for ~15 weeks; 5 weeks longer than in Suzuki and Yamasaki [30]. Nonetheless, molt stages are identical in the two species, demonstrating that they are a stable and robust temporal measurement of post-embryonic development.

**Fig 4:**
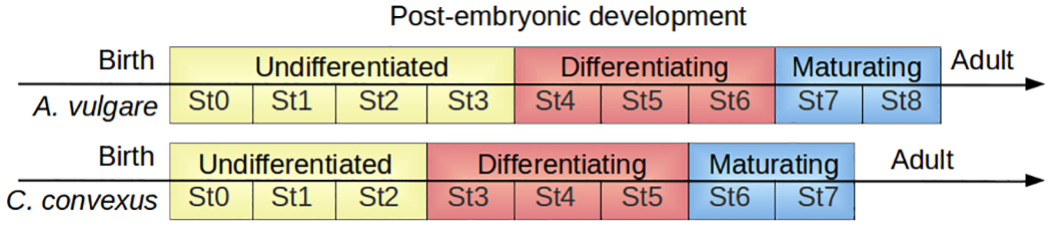
Sexual differentiation timing in *A. vulgare* and in *C. convexus*. Gonad status is indicated above the stage number. *C*. *convexus* sexual differentiation begins one stage earlier than that of *A*. *vulgare* and lasts for the same number of stages.

The most significant difference observed between *C*. *convexus* and *A*. *vulgare* is that *C*. *convexus* post-embryonic development lasts for 7 stages whereas that of *A*. *vulgare* lasts for 8 stages (Fig 4) [30]. In *C*. *convexus*, undifferentiated gonads start to differentiate at stage 3, one stage earlier than in *A*. *vulgare* (Fig 4) [30]. Gonad differentiation continues until stage 5 when testes are filled with mature sperm, which occurs at stage 6 in *A*. *vulgare* [30]. Finally, differentiated gonads mature during stages 6 and 7 in *C*. *convexus*, while this occurs during stages 7 and 8 in *A*. *vulgare* (Fig 4) [30]. Hence, regarding gonad development, *C*. *convexus* sexually differentiates earlier (one stage shift) than *A*. *vulgare*, during the same number of stages (Fig 4). However, differentiation of female genital apertures and male copulating pleopods occurs at stage 5 in both species [42].

Another study previously described the beginning of gonad morphological differentiation in two other Oniscidea species [41]. In *Porcellio dilatatus*, gonad morphology is similar to *C*. *convexus* and *A*. *vulgare*, while *Helleria brevicornis* has a different gonad morphology. Indeed, *H*. *brevicornis* male gonads also presents three testes implanted on the seminal vesicle, but only harbor two androgenic glands, one attached in the middle of the seminal vesicle and the other one on the *vas deferens* [41]. Sexual differentiation of *P*. *dilatatus* begins at the same stage as *C*. *convexus* (stage 3), whereas *H*. *brevicornis* begins one stage earlier, at stage 2 [41]. Altogether, these results indicate that there is a fair amount of diversity in sexual differentiation timing in terrestrial isopods.

### Vertical Transmission of *w*VulC in *C*. *convexus*

We detected *Wolbachia* in ~16% of descendants produced by transinfected *C*. *convexus* females, showing that *w*VulC had been vertically transmitted from the maternal lineage. However, *w*VulC transmission rate in the heterologous host *C*. *convexus* (~16%) is currently lower than in its natural host *A*. *vulgare* (~82%) [21]. Transinfection success with the native *w*Con strain would likely be higher, but the low *w*VulC transmission rate in *C*. *convexus* is not surprising as it concerns the first generation of infection in the host, produced by horizontally infected mothers. Indeed, the host and the bacteria are not adapted to each other as it is the first generation of vertical transmission. After artificial horizontal transfer, it is common that the symbiont is lost through generations [43][44][45][46], although sometimes the symbiont can durably settle in the host [47][48][49][50]. Even with a low transmission rate, these results indicate that *w*VulC can pass two out of three filters that allow the bacteria to settle durably in the new host after horizontal transfer [51]. First, *w*VulC maintains itself in *C*. *convexus*, including in germinal cells [1]. Second, *w*VulC is transmitted to the next generation. It is noteworthy that *w*VulC does not invade *Armadillo officinalis*, as transinfected *Wolbachia* seem to totally disappear [35]. Moreover, *w*VulC also failed to invade a new species when transferred to *P*. *dilatatus* due to the death of the recipient host after an excessive autophagic reaction [52][53]. However, previous experimental transfers showed that *w*VulC can also efficiently invade *A*. *nasatum* and be transmitted to progenies whose males are feminized by the bacteria [34]. Therefore, the third filter for *w*VulC to pass in *C*. *convexus* would be the ability to induce feminization in its new host, as this would increase bacterial transmission.

### Feminization Effect of *w*VulC in *C*. *convexus*

In terrestrial isopods, *w*VulC injection in males strongly disturbs male secondary sexual characters, leading to intersexes in several recipient hosts, including *C*. *convexus* [35][36]. In this study, all *C*. *convexus* transinfected adult males in which *Wolbachia* was detected presented hypertrophied androgenic glands. In contrast, only two thirds of them harbored one or two female genital apertures, suggesting a gradual feminizing effect of the bacteria. As hypothesized by Rigaud *et al*. [27], feminizing efficiency might rely on *Wolbachia* density. Indeed, transinfection of serial dilutions of feminizing *Wolbachia* extracts in *A*. *vulgare* adult males showed that the more the extract was diluted, the less efficient feminization of males became [27].

We also investigated feminization effect of inherited *w*VulC in progenies of transinfected *C*. *convexus* mothers. We showed that from the very first generation of *C*. *convexus* which has vertically inherited *Wolbachia*, *w*VulC seems to be able to induce a sex-ratio bias towards females (Table 2). However, the correlation of *Wolbachia* presence only in females and intersexes is not perfect. Infected males harboring no morphological abnormalities have never been observed in *A*. *vulgare* natural populations hosting *w*VulC [12][21]. Nonetheless, terrestrial isopod males can be infected with feminizing strains of *Wolbachia* in *Oniscus asellus* and *Porcellionides pruinosus* [54][55][56]. This change of feminization efficiency induced by *w*VulC is not surprising as the extended phenotype induced by *Wolbachia* in their native host is rarely conserved in a transinfected host, especially for feminization [34][57]. Thus, perfect feminization may require a specialized relationship between the host and the symbiont as it requires fine manipulation of host physiology.

Sometimes, in *A*. *vulgare*, incomplete feminization can occur as demonstrated by the presence of intersexes [12][33]. In *C*. *convexus*, about 10% of the progenies produced by transinfected mothers were intersexes. It is noteworthy that intersexes have never been observed; neither in controls, nor in uninfected *C*. *convexus* lines raised in our laboratory for almost 20 years. Intersexes are expected to be derived from incomplete feminization caused by *Wolbachia* infection, although we detected *w*VulC by PCR in only one intersex. If so, it is possible that the transmission rate we calculated (~16%) is underestimated.

The feminizing effect of *w*VulC can be directly observed in *C*. *convexus* juveniles produced by transinfected mothers, as attested by the altered gonads with intersexual phenotype during post-embryonic development. This suggests that *w*VulC alters gonad differentiation of the new host, as in *A*. *vulgare* [58]. As fewer normal males (16.6%) than expected (48.3%) were observed in these *C*. *convexus* juveniles, we assumed that the 14 individuals with intersexual gonads are genetic males that are being feminized (as expected in the natural host *A*. *vulgare*) [2].

Sex-ratio bias towards females in infected progenies, intersexual phenotypes in progenies throughout development, and appearance of female secondary sexual characters in transinfected adult males, together clearly indicate that *w*VulC has a feminizing effect in *C*. *convexus*. Feminization occurs after vertical inheritance, despite an earlier sexual differentiation when compared to *A*. *vulgare*. This suggests that feminization mechanisms induced by *w*VulC are not highly specialized with respect to host sexual differentiation [26]. The absence of coevolution between *w*VulC and its heterologous host *C*. *convexus* may explain a lower transmission rate and a less efficient feminizing effect than in the native host of *w*VulC. We are currently obtaining additional generations of *w*VulC-infected *C*. *convexus* isopods using crosses involving infected female individuals, which should select for *Wolbachia* genotypes best adapted to the new host. The establishment of a stable line of *C*. *convexus* infected with the feminizing *w*VulC strain constitutes an asset to study the molecular mechanisms of feminization. Indeed, it will allow further comparative studies between the natural and well-studied model *w*VulC/*A*. *vulgare* and the new model *w*VulC/*C*. *convexus*, which has different sexual differentiation timing. Such an experimental system will enable discrimination between the confounding effects of the feminizing action of *w*VulC linked to sexual differentiation from an alternative action unrelated to feminization, but fortuitously occurring during sexual differentiation.
